# Time-Spectral based Polarization-Encoding for Spatial-Temporal Super-Resolved NSOM Readout

**DOI:** 10.1038/s41598-019-49721-w

**Published:** 2019-09-11

**Authors:** Matityahu Karelits, Yaakov Mandelbaum, Zeev Zalevsky, Avi Karsenty

**Affiliations:** 1 Lev Academic Center, Dept. of Applied Physics/Electro-Optics Eng., Advanced Laboratory of Electro-Optics (ALEO), Jerusalem, 9116001 Israel; 2 Faculty of Engineering, Bar-Ilan University, Ramat Gan, 5290002 Israel; 3 The Nanotechnology Center, Bar-Ilan University, Ramat Gan, 5290002 Israel; 4 The Nanotechnology Educational and Research Center, Lev Academic Center, Jerusalem, 9116001 Israel

**Keywords:** Nanophotonics and plasmonics, Imaging and sensing

## Abstract

Detection of evanescent waves through Near-field Scanning Optical Microscopy (NSOM) has been simulated in the past, using Finite Elements Method (FEM) and 2D advanced simulations of a silicon Schottky diode, shaped as a truncated trapezoid photodetector, and sharing a subwavelength pin hole aperture. Towards enhanced resolution and next applications, the study of polarization’s influence was added to the scanning. The detector has been horizontally shifted across a vertically oriented Gaussian beam while several E-field modes, are projected on the top of the device. Both electrical and electro-optical simulations have been conducted. These results are promising towards the fabrication of a new generation of photodetector devices which can serve for Time-Spectral based Polarization-Encoding for Spatial-Temporal Super-Resolved NSOM Readout, as developed in the study.

## Introduction

There is a broad literature on the role of a metallic layers put on top of Near-field Scanning Optical Microscopy (NSOM) probes^1–4^ to enhance the resolution with use of plasmonic effects. As it is well known, the Transverse Electric (TE) polarization cannot excite a propagating surface plasmon^5,6^. In order to excite a Propagating Surface Plasmon (PSPP), as opposed to the plasmons which play a role in surface enhancement, an electric field component perpendicular to the surface is required. This is necessary in order to create a dipole polarization density P, with component normal to the surface, resulting in a bound charge surface density (on the surface). This in turn leads to the discontinuity in the electric field central to the PSPP. For the out of plane (TE) polarization, the incident electric field is parallel to the surface and so a PSPP can’t be excited. However, it is correct that, if the direction of propagation of the incident wave has an out of plane component, a TE wave can indeed excite a PSPP: more precisely the distinction between Transverse Electric (TE) and Transverse Magnetic (TM) blurs as the so-called TE polarization must be orthogonal to the direction of propagation and hence is no longer exactly transverse. A component of the electric field then exists, transverse to the metal plane, which is a condition for the excitation of a PSPP as explained above. As the out-of-plane angle increases this component increases and hence the importance of a PSPP may be more significant. On the other hand as the out-of-plane angle increases, the component of the E-field along the device aperture decreases and hence the optical absorption decreases. Thus out-of-plane propagation can be deemed less significant. It is possible to simulate out-of-plane propagation direction without requiring a full three-dimensional model, however there are sometimes strong logistics limitations with a 3D model. A boundary condition which describes a real metal is the Impedance Boundary Condition (IBC). IBC’s can be used in numerical studies to simulate the propagation of PSPP’s.

**Figure 1:**
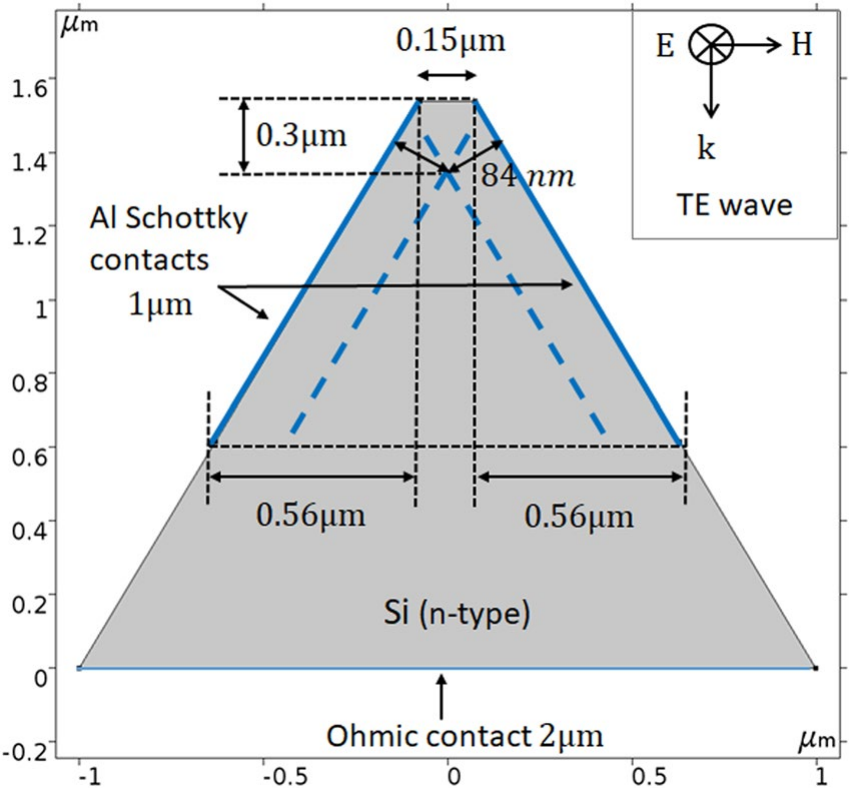
Comsol 2D simulation of the NSOM photodetector structure. The depletion layer boundaries are marked by blue dashed lines for a given bias of −0.5 V applied at the Schottky contact. The TE wave components are indicted in the insert.

**Figure 2:**
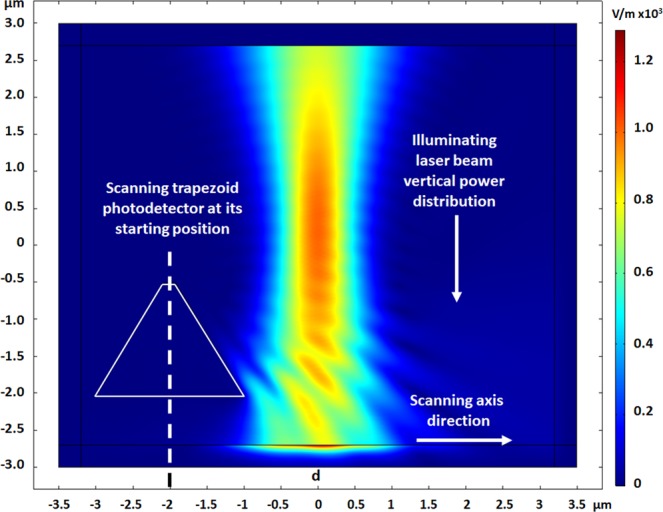
Initial conditions of the scanning process, with the device located on the left side of the laser beam, incoming from above. The Gaussian laser beam has a Full Width at Half Maximum of 0.69 μm. The distance from the top center of the photodetector to the beam center is 2 μm.

**Figure 3:**
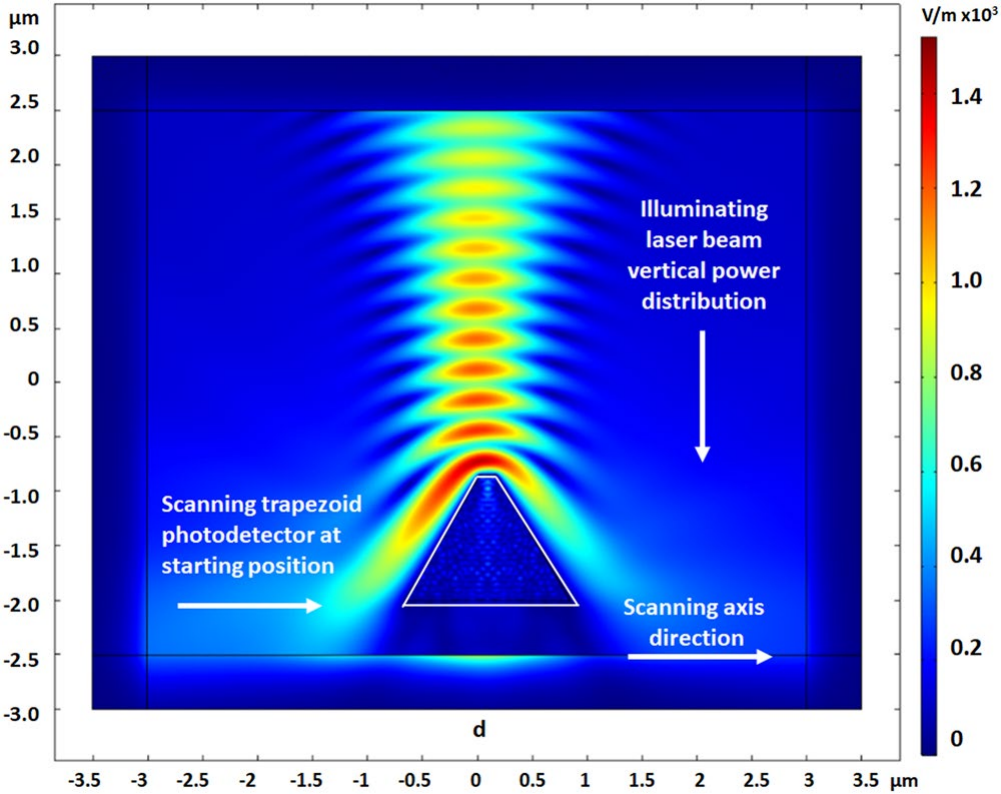
Simulation result of the beam scanning, using a shifted trapezoid moving horizontally under the vertical illumination. The distance from the top center of the photodetector to the beam center is 0.5 μm.

In this research, we present a 2D model for the study of the influence of such E-field with different polarizations. The electro-optical scanning of a vertical polarized laser beam is presented here, using a shifted photodetector (Fig. 1) sharing a 2D trapezoid shape and a sub-wavelength aperture, in front of an illumination beam (Figs 2 and 3). As presented in the article, the study consisted in varying the polarization critical parameter of the Gaussian beam in *X* and *Y* directions, demonstrating strong differences. Additional and complementary studies present the polarization phenomena as a function of important parameters such as: *d* the scanning distance, *E*_*X*_ and *E*_*Y*_ the electric field in polarization directions, *λ* the wavelength, and *A* the photodetector aperture. All parameters used in the simulations are summarized in Table 1. In order to show preliminary experimental validation, an NSOM/AFM combined tip was fabricated. An AFM tip served for the fabrication of a photodetector on the tip such that the photonic readout could be provided directly. Short fabrication process and functionality measurement setup are also presented. Such a forecast analysis can serve, among others, for several applications, and in particular for Time-Spectral based Polarization-Encoding for Spatial-Temporal Super-Resolved NSOM Readout.

**Table 1:** Summarizing table of the used parameters.

Parameter	Parameters definition	Values and units
**Device parameters:**		
*N*_*D*_	n-doped channel donors (P) concentration	10^16^ cm^−3^
*Φ*_*m*_	Metal work function (Al)	4.72 eV
*A*	Top aperture diameter	150 nm
*L*_*S*_	Al Schottky contact length	1 µm
*L*_*O*_	Ohmic contact width	2 µm
*H*	Trapezoidal structure height	1.54 μm
**Setup used parameters:**		
*W*	Beam waist	0.5 µm
*R*	Radiant intensity	0.15 W/m^2^
*λ*_*scanning*_	Wavelength of the laser beam	550 nm
*FWHM*	Full Width at Half Maximum of the laser beam	0.69 μm
*λ*_*range*_	Extended range of wavelengths	400 nm to 1100 nm
*d*	Longest scanning distance	−3 µm to 3 µm
*V*_*b*_	Bias voltage	−0.5 V
*a*	Distance from photodetector top to beam center	0.5 µm
**Measured parameters:**		
*E*_*X*_	Electric Field with Polarization in X direction	V/m
*E*_*Y*_	Electric Field with Polarization in Y direction	V/m
*I*_*X*_	Measured photo-induced current in X direction	pA
*I*_*Y*_	Measured photo-induced current in Y direction	pA

## Simulation Method and Results

A detailed description of the simulation was presented in^7^ for the three-dimensional pyramidal device. The two-dimensional simulation is similarly performed; hence the technical description is brought in this section. Here we discuss a few selected points which are specific to the two-dimensional simulation.

### Metallic layer and boundaries

As shown in Fig. 1, the external walls of the photodetector are composed of a coated layer of aluminum. When looking at the upper section of the device, one can observe that this layer serves as the anode of the Schottky junction. For simulation convenience, a perfect metal was assumed, i.e. a Perfect Electric Conduction (PEC) boundary condition was used, which imposes a Dirichlet condition on the component of the electric field parallel to the surface. The metal-semiconductor junction is modeled as a boundary condition, and the metal component itself has no thickness and does not model internal bulk currents.

### Surface polariton-plasmon resonances

TM electromagnetic waves only can excite Surface Polariton-Plasmon (SPP) resonances at the insulator to metal interface. Assuming the modeling of metal boundaries as perfect conductors, this translates into the Dirichlet boundary condition on the electric field: *E*_||_ = 0, and corresponds to zero impedance and vanishing skin depth, so that the electric field is entirely excluded from the interior of the metal. In this limit, the conductance is infinite so that the complex dielectric function is also infinite according the Drude equation^8^:

1 $${\epsilon }={{\epsilon }}_{{\rm{real}}}+j\frac{\sigma }{\omega }$$

The Surface Polariton-Plasmon wave number [5] is then reaching the total wavenumber k:

2 $${k}_{||}=\frac{\omega }{c}\sqrt{\frac{{\epsilon }{{\epsilon }}_{r}}{{\epsilon }+{{\epsilon }}_{0}{{\epsilon }}_{r}}}\mathop{\to }\limits^{{\epsilon }\to \infty }\frac{\omega }{c}\sqrt{{{\epsilon }}_{r}}=n\frac{\omega }{c}=k$$

where ${{\epsilon }}_{r}$ is the dielectric constant of the surrounding dielectric, and *n* the index of refraction. This implies the nullity of the normal component $\,{k}_{\perp }$. Combined with above requirement of electric field orthogonal to the surface, this leads that only beams at perfectly glancing incidence can excite an SPP on the metal surface. The Gaussian beam propagates along the negative y-axis but includes plane wave components along the other direction. These are distributed in k-space (the ‘angular distribution’) with a Gaussian distribution of width inverse to the waist. Due to the sloping sides of the device, glancing incidence, like other off-axis components directions is suppressed. However, the absorbance peak of a surface plasmon is quite narrow [5], with regard to the angle of incidence, even for a real metal with nonzero dissipation. Thus the radiation absorbed in the SPP channel, obtained by integrating the product of the EM wave distribution with the SPP resonant peak, is expected to be quite small. This means that TE polarization becomes the dominant mechanism for electromagnetic interactions for such device architecture.

### 2D simulation conditions and process

Though 3D simulations are ideal, many 2D simulations have been performed in the past due to constraints of computing power^9–13^. Sometimes the applicability of the results was understood to be limited to the qualitative aspects. However in this work we analyze the behavior of a device whose orthogonal dimension is significantly larger than its lateral dimensions, so that a two dimensional model is more than sufficient to simulate its behavior. This detector has a large extent in the z-direction, orthogonal to the plane of the simulation. Its aperture is thus a slit – subwavelength in the plane of the simulation, unconfined in the orthogonal direction. Accordingly, the impinging radiation is incident in the plane of the radiation; the Gaussian beams employed are 2-D Gaussian beams.

The simulation was performed with the Comsol Multi-Physics Software Package^14^, which uses the Finite Elements Method (FEM) for special devices design^15^. The simulation couples the Semiconductor and the Wave Optics modules using the Optoelectronics interface. The Semiconductor module computes the electronic transport and charge distribution within the silicon using the drift-diffusion equations for the electron and hole densities in the semiconductor coupled to Poisson’s equation for the electric ‘band-bending’ potential. These are augmented by statistical mechanics to describe the effect of doping on the carrier charge population. Light-matter interactions, described ahead, occur primarily within the Schottky junction which develops between the semiconductor bulk and metal surface. The junction is a depletion layer which develops in the semiconductor due to the difference in Fermi levels between the two materials. The work function of the metal, the electron affinity of the semiconductor and the doping level are input parameters. In addition the phenomenon of thermionic emission from the metal to the semiconductor is built as a boundary condition on the drift-diffusion current, which determines the boundary conditions on the charge distribution.

The material chosen for the interior of the device was Silicon, n-doped to a donor concentration N_D_ = 10^16^ cm^−3^ using Analytic Doping. Trap-Assisted Recombination was activated, with default values. The sides of the device serve as the Schottky junction. This is realized using the Metal Contact boundary condition with the Rectifying Junction option selected, together with ‘Thermionic Emission’. The material chosen for the model was Aluminum, which sets the work function, and the external bias was set to the appropriate voltage. Together these determine the boundary condition for the electric potential $\varphi$:

3 $${qV}={{E}}_{{F},{\rm{m}}}={q}\varphi -{\Phi }_{{m}}$$

where Φ_m_ is the i.e. the metal work function, and *V* is the external bias. The Schottky junction only extends along the upper section of the device. The rest of the exterior is given an Insulating boundary condition. This defines the boundary conditions relative to the Semiconductor module.

The wave optics module simulates the propagation of the electromagnetic wave using Maxwell’s equations. On the entire outer surface of the device (excluding the ends) a perfect electric conduction boundary condition was used, which imposes a Dirichlet condition on the component of the electric field parallel to the surface. These effects give a perfectly reflecting boundary, i.e. a perfect metal. This is a good approximation, as the entering electromagnetic wave decays entirely in the aluminum before penetrating through to the silicon in the interior. In order to simulate photo-detection ‘Optical Transitions’ was activated in the interior. This adds a photo-generation source term to the convection-diffusion equations, which is proportional to the intensity of the electromagnetic field. The interaction between the radiation and the silicon – photo-detection – involves indirect and direct inter-band transitions. The precise form of this term is determined by Fermi’s Golden Rule, together with an empirical model for indirect absorption in Silicon, based on an empirical model of Green and Keevers^16^.

The incoming optical excitation is effected at the upper surface using a Port boundary condition with the relevant value of the Electromagnetic intensity. The lower surface is given an open boundary condition using the Scattering Boundary Condition selection. Finally with regards to the electric potential the upper surface is given an insulating boundary condition, while the lower surface is defined as a Metal Contact boundary condition with the Ohmic Contact option.

### 2D scanning of laser beam illumination

The NSOM detector is simulated in two dimensions as a trapezoidal structure of 1.54μm height, 2μm bottom width, and 150 nm top aperture diameter. If used in the past for slit imaging^17^, it is now used for polarization study. As shown in Figs 2 and 3, the device – identified by its trapezoid silhouette – is shifted horizontally across the vertical projected Gaussian laser beam of 550 nm wavelength having a 0.69μm Full Width at Half Maximum (FWHM). The color scale indicates the beam intensity as the electric field value (V/m). In Fig. 2, the device is presented at its starting point, on the left side of the beam. The scanning proceeds when the device is shifting to the right direction. In Fig. 3, the device’s position approaches the center of the beam where it absorbs part of the intensity. A visual demonstration of the scanning process, with the device located on the left side of the laser beam, incoming from above, is presented in the linked animated GIF.

Our analysis consists in varying several parameters in order to optimize the photo current response. The metal contact is 1 µm height from the top aperture (both sides of the trapezoid). The ground electrode is at the bottom of the structure. The first class of parameters we will consider is the device intrinsic specifications like the silicon doping level and the metal work function. Then we will consider the influence of external parameters like the bias voltage, the radiant intensity and the laser wavelength.

### Polarization aspects

**Figure 4:**
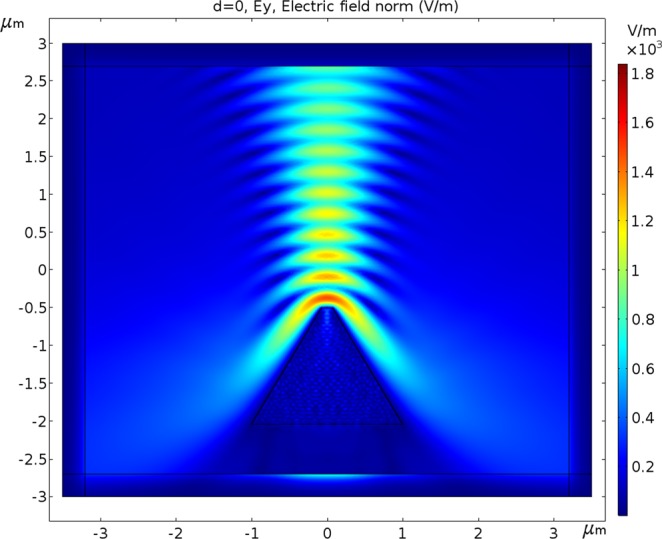
The scanned Gaussian beam has a Full Width at Half Maximum of 1.1 μm. The distance from the top center of the photodetector to the beam waist is 0.5 μm. Doping is 10^16^ cm^−3^, work function is 4.72 eV (Al), bias voltage is −0.5 V, λ is 550 nm with polarization direction Y (out of plane) and radiant intensity is 0.15 W/m^2^.

**Figure 5:**
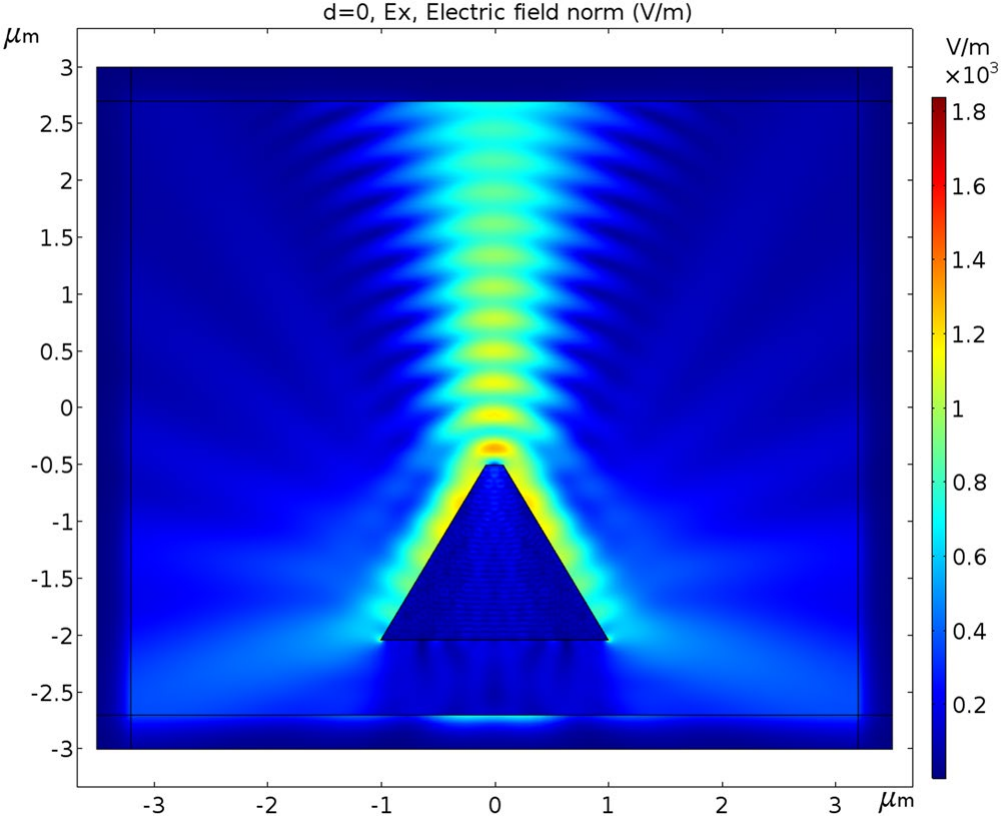
The scanned Gaussian beam has a Full Width at Half Maximum of 1.1 μm. The distance from the top center of the photodetector to the beam waist is 0.5 μm. Doping is 10^16^ cm^−3^, work function is 4.72 eV (Al), bias voltage is −0.5 V, λ is 550 nm with polarization direction X (out of plane) and radiant intensity is 0.15 W/m^2^.

In Fig. 4, the polarization direction of the Gaussian beam is Y, while in Fig. 5, the polarization direction of the Gaussian beam is X. In both the simulations, the device’s position is under the center of the beam during scanning from left to right.

**Figure 6:**
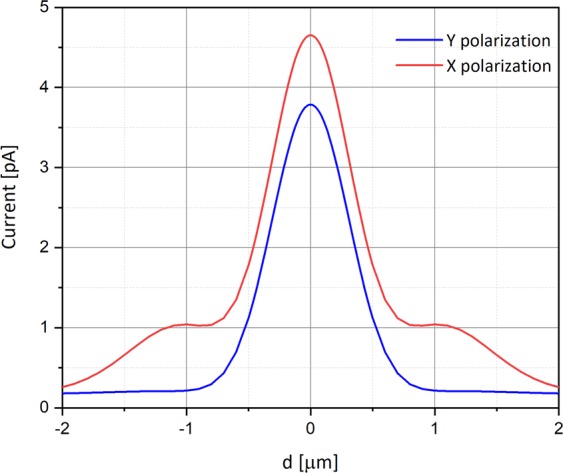
Current as a function of the photodetector’s position [in μm], relative to the beam center, for X and Y polarizations. The simulation was performed under the following conditions: Doping is 10^16^ cm^−3^, λ is 550 nm, bias voltage is −0.5 V, radiant intensity is 0.15 W/m^2^ and work function is 4.72 eV (Al).

Figure 6 shows the current measured in the simulation at the metal electrode by scanning the detector across the laser beam as described above. The varying parameter is the polarization of the Gaussian beam. The simulation was performed under the following conditions: The metal work function is 4.72 eV (Al), wavelength λ is 550 nm, the bias voltage is −0.5 V, and the electric field is 10^3^ V/m equivalents to a radiant intensity of 0.15 W/m^2^.

**Figure 7:**
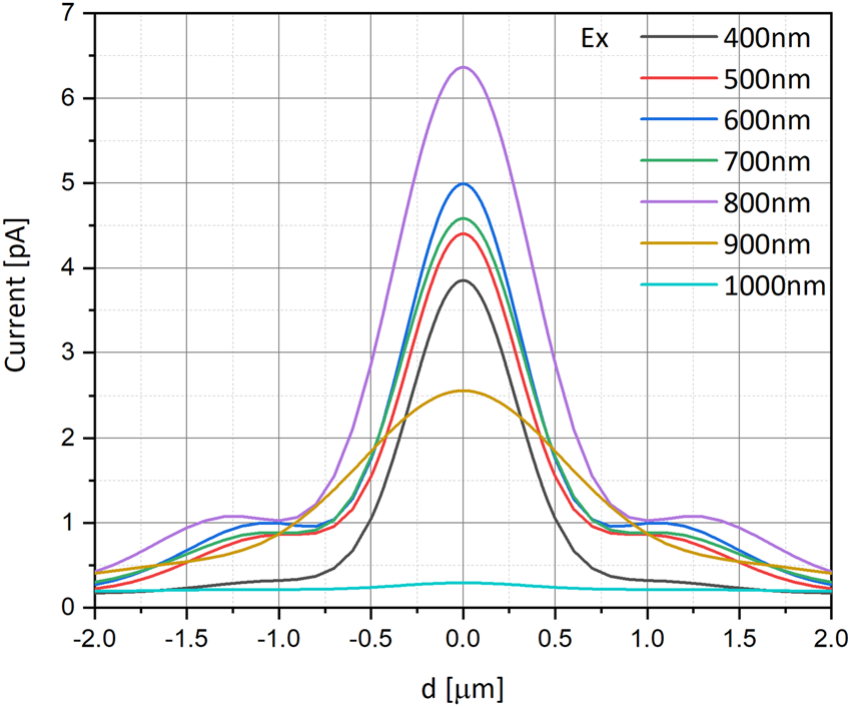
Current as a function of the photodetector’s position [in µm] for X polarization, relative to the beam center, for several wavelength values. The simulation was performed under the following conditions: The top aperture diameter is 150 nm, the doping is 10^16^ cm^−3^, the work function is 4.72 (Al), the voltage bias is −0.5 V, and the radiant intensity is 0.15 W/m^2^.

**Figure 8:**
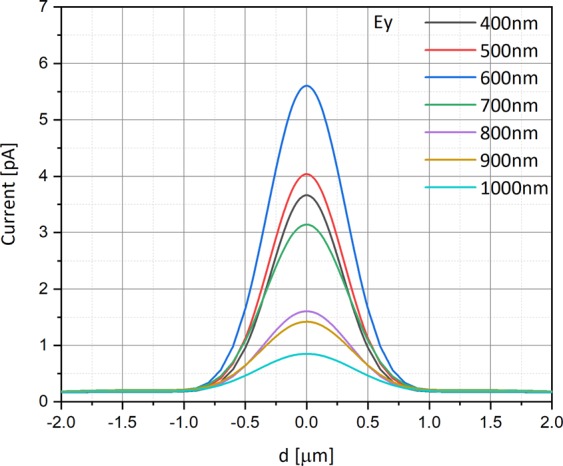
Current as a function of the photodetector’s position [in µm] for Y polarization, relative to the beam center, for several wavelength values. The simulation was performed under the following conditions: The top aperture diameters is 150 nm, the doping is 10^16^ cm^−3^, the work function is 4.72 eV (Al), the voltage bias is −0.5 V, and the radiant intensity is 0.15 W/m^2^.

Figures 7 and 8 respectively show the current measured in the simulation at the metal electrode by scanning the detector across the laser beam as described above. The varying parameter this time is the wavelength ranging between 400 nm and 1100 nm, by steps of 100 nm, and the scanning is done for both the polarizations. The simulation was performed under the following conditions: The metal work function is 4.72 eV (Al), the bias voltage is −0.5 V, and the electric field is 10^3^ V/m equivalents to a radiant intensity is 0.15 W/m^2^.

**Figure 9:**
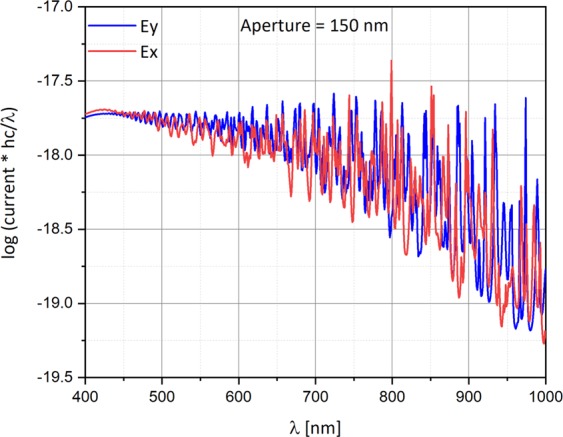
Semi-logarithmic graph current with photon energy weighting as a function of the wavelength for both polarizations. The top aperture diameter is 150 nm. The simulation was performed under the following conditions: The silicon doping is 10^16^ cm^−3^, the work function is 4.72 eV (Al), the voltage bias is −0.5 V, and the radiant intensity is 0.15 W/m^2^.

**Figure 10:**
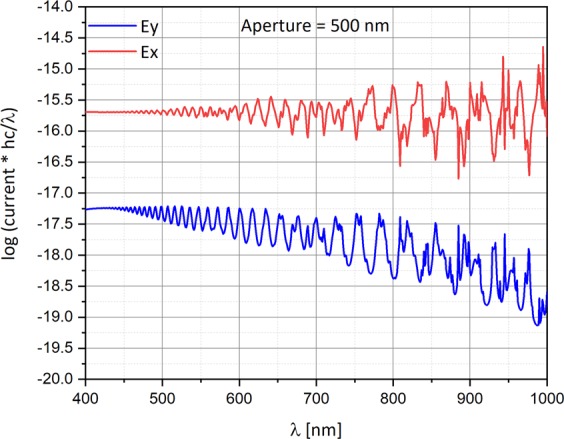
Semi-logarithmic graph current with photon energy weighting as a function of the wavelength for both polarizations. The top aperture diameter is 500 nm. The simulation was performed under the following conditions: The silicon doping is 10^16^ cm^−3^, the work function is 4.72 eV (Al), the voltage bias is −0.5 V, and the radiant intensity is 0.15 W/m^2^.

The behavior of the photo-current with photon energy weighting as a function of the wavelength, this time for several aperture diameters (A = 150 nm, and A = 500 nm) and both polarizations, is respectively presented in Figs 9 and 10, while the trapezoid is situated under the center of the Gaussian beam. The simulation was performed under the following conditions: The metal work function is 4.72 eV (Al), the bias voltage is −0.5 V, and the electric field is 10^3^ V/m, equivalent to a radiant intensity is 0.15 W/m^2^.

While for an aperture of 150 nm (Fig. 9) E_x_ and E_y_ are almost matched, for an aperture of 500 nm (Fig. 10), the two polarizations are separated.

## Temporal Super Resolution Via Time-Spectral Polarization Encoding

Due to the high spatial localization of the scanning probe, the NSOM probe can produce high spatial sub-wavelength resolution. However, it is well known that eventually the resolution limit is directly associated to the Signal To Noise ratio (SNR)^18^. This is obvious since the localization capability and precision is directly related to the SNR^19^. Thus, it could be advantageous to perform the temporal readouts coming from the probe at low temporal frequency, i.e. to sample fast but to average many samples in order to enhance the obtainable SNR. However, such temporal averaging reduces the temporal resolution which, given known scanning velocity V directly affects the spatial resolution dx:

4 $$dx=Vdt$$

Where *dt* is the reduced temporal resolution after performing the temporal averaging.

As seen in e.g. Figure 6 there is a position dependent ratio between the two principle polarizations. According to Figs 7 and 8 this ratio is also wavelength dependent. Thus, if for instance we aim to scan a 2D structure that in one dimension is subwavelength and in the other is not then the back reflected information will have strong polarization along the sub wavelength axis^20^.

Please note that in the mathematical derivation below we do not address at all the scanning strategy, which could be an interesting direction by itself, to perform the polarization related encoding/decoding process. However, in the current manuscript we did not assume any special scanning strategy. We just assume that a temporal polarization changing signal *p*_*s*_(*t*) is obtained during the scanning process and we also assume that if during the scanning and the spatial mapping process, the tip is also slightly rotated or/and the illumination wavelengths are slightly changed then for uniform object a predicted time-wavelength dependent change in the polarization ratio is expected. This is our encoding pattern which we notate by *p*_*c*_(*t*,). We assume that the encoding pattern has fast temporal fluctuations, as fast as the temporal information in the signal *p*_*s*_(*t*). The readout is low-passed in time domain. We notate this low passing time response as *h*(*t*). Thus, the readout obtained via our proposed time scanning tip equals to:

5 $$r(t,\lambda )={\int }_{t\text{'}}({p}_{s}(t^{\prime} ){p}_{c}(t^{\prime} ,\lambda ))h(t-t^{\prime} )dt^{\prime} $$

Where the temporal length of h(t) is Δ*t* being the time averaging needed for enhancing the SNR but which also damages the temporal and therefore the spatial resolution. We will now perform a decoding of the captured low-passed readout. The decoding pattern is equal to the a priori known encoding pattern:

6 $$d(t)={\int }_{\lambda }r(t,\lambda ){p}_{c}^{\ast }(t,\lambda )d\lambda $$

We will substitute the expression for r(t, *λ*) into our last integral and obtain:

7 $$d(t)={\int }_{t^{\prime} }{\int }_{\lambda }({p}_{s}(t^{\prime} ){p}_{c}(t^{\prime} ,\lambda ))h(t-t^{\prime} ){p}_{c}^{\ast }(t,\lambda )dt^{\prime} d\lambda $$

We also know that the wavelength dependence can be designed to be highly orthogonal (dependent on the proper design of our configuration as seen in Figs 6–8) and thus:

8 $${\int }_{\lambda }{p}_{c}(t^{\prime} ,\lambda ){p}_{c}^{\ast }(t,\lambda )d\lambda =\delta (t-t^{\prime} )$$

Note that the wavelength dependent averaging process comes simply by having the readout via the same tip from which the illumination (encoding) took place. So, all we need is a detector that collects (integrates) the information over the relevant spectral range of illumination.

Thus, using this expression in our previous equation yields the resulted readout to be:

9 $$d(t)={\int }_{t}{p}_{s}(t^{\prime} )\delta (t-t^{\prime} )h(t-t^{\prime} )dt^{\prime} =h(0){p}_{s}(t)$$

Since h(0) is only a constant, we are basically able to reconstruct the temporal polarization changing signal *p*_*s*_(*t*) without being lowpassed by the time averaging operation of the probe.

## Preliminary Experimental Validation

**Figure 11:**
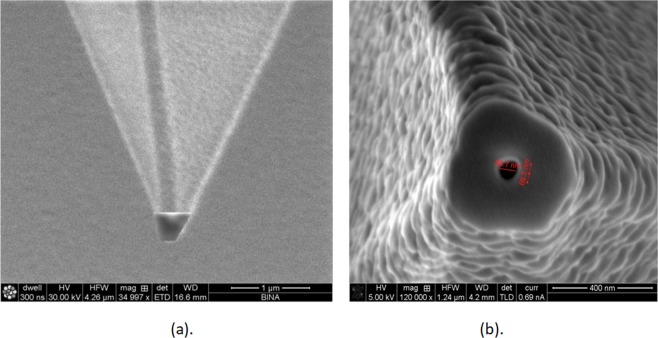
The fabricated tip. (**a**) SEM view of the tip area using the FIB system after its ablation. (**b**) SEM pictures of the tip after drilling and platinum deposition needed to obtain conductance of the fabricated photo detector. The view of a tip contact diameter of ~400 nm and aperture diameter of 68.7 nm.

In order to show preliminary experimental validation, we have fabricated an NSOM/AFM combined tip. We took an AFM tip and fabricated a photodetector on the tip such that the photonic readout could be provided directly. In Fig. 11 we show the fabrication process where we present SEM view of the tip area after processing it with FIB system after its ablation, and then SEM picture of the AFM tip after drilling and Platinum deposition needed to generate conductance.

**Figure 12:**
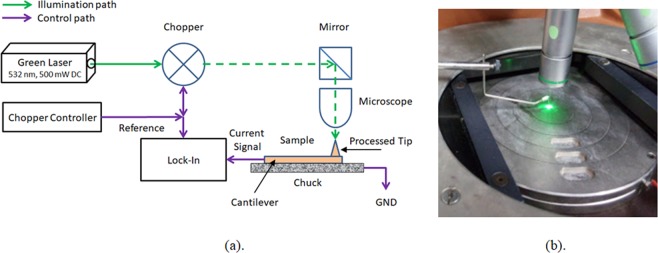
(**a**) Experimental set-up of the NSOM measurement. (**b**) Processed illuminated sample in the set-up experiment.

In order now to verify the good functionality, a classic experimental set-up was designed and tested, as presented in Fig. 12. The sample is placed on a chuck controlled by vacuum. The illumination is done using a green laser (532 nm, 500 mW DC), when the beam optical path is progressing through a chopper and a mirror, then focused on the sample, means the processed tip of the cantilever. Control and measurement roles are enabled using chopper controller and lock-in amplifier. As part of the functionality check, the chopper was run at several frequencies (365 Hz, 770 Hz), and several processed samples were used for measurements in two configurations: Laser Off and Laser On (Full Power), as summarized in Table 2. A broken tip was used for reference when compared to the samples.

**Table 2:** Summarizing table of the electrical measurements as a function of the laser illumination.

Probe dia.	400 nm	200 nm	Reference (No cantilever)
Laser OFF	280 pA	200 pA	278 pA
Laser ON	254 nA	190 nA	312 pA

The samples were chosen with large probe diameter to first assure a good light absorption. As presented in Table 2, there is a significant difference in measured currents when laser is set Off or On, demonstrating the functionality of the device.

Those are just preliminary experimental results, involving both fabrication and functionality testing, and they still do not dmeonstrate how the polarization dependence can produce super resolved readouts and those will be our tasks in our future research. However, looking at all above results, it appears that there is a clear sensitivity to the laser illumination and there is a clear apparent discrimination between reference (no cantilever) and processed tips.

## Conclusions

In this article, the electro-optical scanning of a vertical polarized laser beam is presented, using a shifted photodetector with a sub-wavelength aperture, in front of an illumination beam. The study consisted in varying polarization modes critical parameter. Clear differences have been observed between sub-wavelenght (150 nm) and wavelenght (500 nm) apertures. If several studies presented some ideas of photodetectors for NSOM purpose, this study enables an easy forecast of the expected behavior of the device, as a function of the changed parameters. In addition to the numerical and diverse studies, and until full experimental validation in the future, preliminary results of processing and functionality measurements are presented, with the fabrication of an NSOM/AFM combined tip. One can assume that a new generation of photodetectors, serving for Time-Spectral based Polarization-Encoding for Spatial-Temporal Super-Resolved NSOM Readout, can make the difference.

## Supplementary information

A visual demonstration of the scanning process, with the device located on the left side of the laser beam, incoming from above, is presented in the linked animated GIF.  Animated GIF
