# Supplementary figures and images for: Time-Spectral based Polarization-Encoding for Spatial-Temporal Super-Resolved NSOM Readout
Source: Sci Rep. 2019 Sep 11;9:13089. doi: 10.1038/s41598-019-49721-w (PMC6739407; doi:10.1038/s41598-019-49721-w)

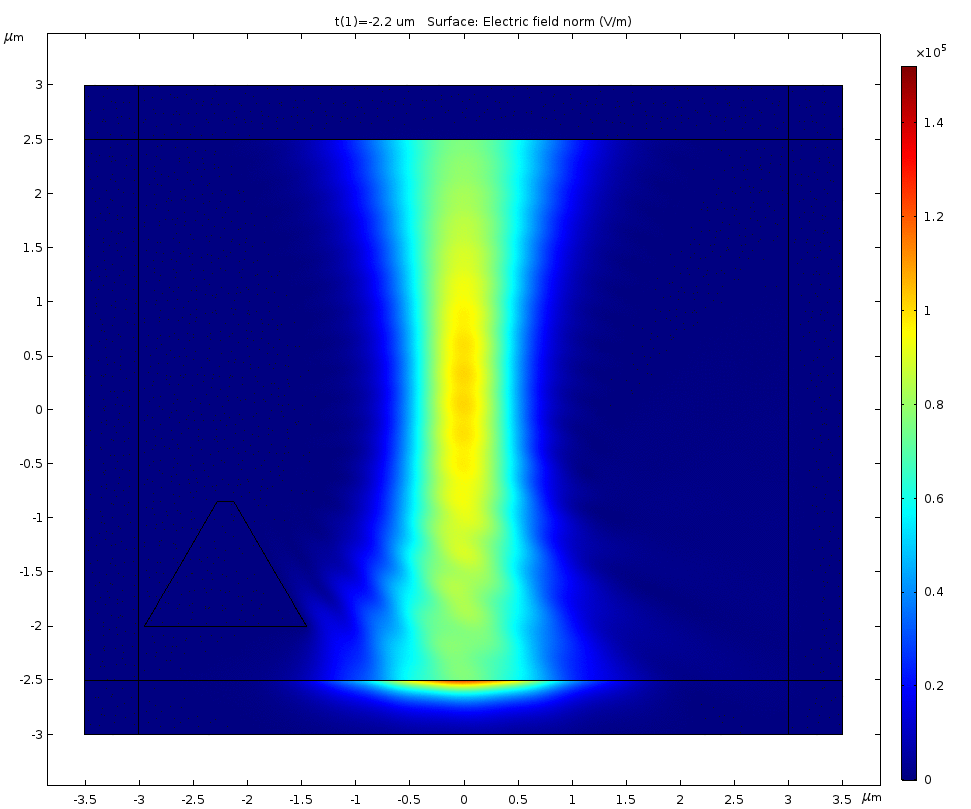

Supplement: Supplementary file 1 — A visual demonstration of the scanning process, with the device located on the left side of the laser beam, incoming from above, is presented in the linked animated GIF. Animated GIF [file 41598_2019_49721_MOESM1_ESM.gif]
